# Upstream trophic structure modulates downstream community dynamics via resource subsidies

**DOI:** 10.1002/ece3.3144

**Published:** 2017-06-15

**Authors:** Eric Harvey, Isabelle Gounand, Chelsea J. Little, Emanuel A. Fronhofer, Florian Altermatt

**Affiliations:** ^1^ Department of Evolutionary Biology and Environmental Studies University of Zurich Zürich Switzerland; ^2^ Department of Aquatic Ecology Eawag, Swiss Federal Institute of Aquatic Science and Technology Dübendorf Switzerland

**Keywords:** cross‐ecosystem subsidies, directional flows, meta‐ecosystems, river ecosystems

## Abstract

In many natural systems, the physical structure of the landscape dictates the flow of resources. Despite mounting evidence that communities’ dynamics can be indirectly coupled by reciprocal among ecosystem resource flows, our understanding of how directional resource flows might indirectly link biological communities is limited. We here propose that differences in community structure upstream should lead to different downstream dynamics, even in the absence of dispersal of organisms. We report an experimental test of the effect of upstream community structure on downstream community dynamics in a simplified but highly controlled setting, using protist microcosms. We implemented directional flows of resources, without dispersal, from a standard resource pool into upstream communities of contrasting interaction structure and then to further downstream communities of either one or two trophic levels. Our results demonstrate that different types of species interactions in upstream habitats may lead to different population sizes and levels of biomass in these upstream habitats. This, in turn, leads to varying levels of detritus transfer (dead biomass) to the downstream communities, thus influencing their population densities and trophic interactions in predictable ways. Our results suggest that the structure of species interactions in directionally structured ecosystems can be a key mediator of alterations to downstream habitats. Alterations to upstream habitats can thus cascade down to downstream communities, even without dispersal.

## 1 INTRODUCTION

In many natural systems, the physical structure of the landscape dictates the flow of organisms and resources. Previous work has shown that directionally biased movement of organisms can have significant effects on species coexistence (Levine, 2003; Lutscher, McCauley, & Lewis, 2007; Lutscher, Pachepsky, & Lewis, 2005; Salomon, Connolly, & Bode, 2010), metapopulation dynamics (Fronhofer & Altermatt, 2017) and stability (Elkin, Possingham, Michalakis, & DeAngelis, 2008; Wang, Haegeman, & Loreau, 2015), and metacommunity structure (Altermatt, Schreiber, & Holyoak, 2011; Bourgeois, González, Vanasse, Aubin, & Poulin, 2016; Dong et al., 2016). Mounting evidence now suggests that communities’ dynamics can be indirectly coupled by the reciprocal spatial exchange of resources, even in the absence of dispersal of organisms (i.e., meta‐ecosystem, Loreau, Mouquet, & Holt, 2003; Gravel, Mouquet, Loreau, & Guichard, 2010; Harvey, Gounand, Ganesanandamoorthy, & Altermatt, 2016). In that context, directionally biased movement of resources is a special case of meta‐ecosystems where spatial feedbacks are only possible in one direction. Such directional flows are especially relevant in ecosystems where the geomorphic structure of the landscapes and physical processes (erosion, gravity, currents) are inherently driving biased movements of resources, such as in rivers, hillslope erosion, and along coastlines or ocean currents. Many of the systems exhibiting this directionality are strongly dependent on external resource inputs, yet, attempts to look at their effects on community dynamics are scarce (but see Polis & Hurd, 1995 on detrital inputs from sea to islands), and contrary to research on reciprocal exchanges (Gounand et al., 2014; Gravel, Guichard, Loreau, & Mouquet, 2010; Leibold et al., 2004), there is no general understanding of how directional resource flows might indirectly link biological communities.

**Figure 1:**
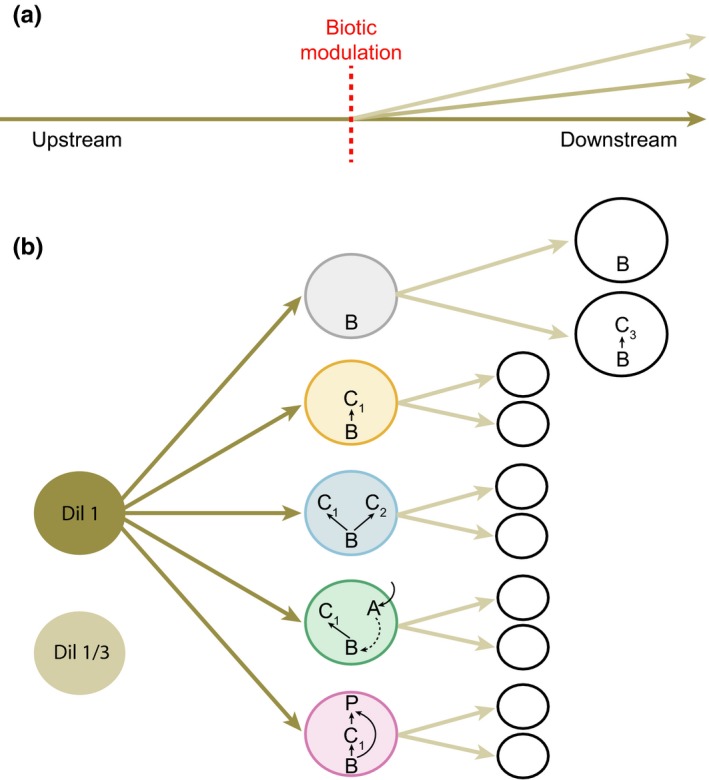
(a) In many ecosystems, resource flow is directionally biased; as they move downstream, these resources will be integrated, processed, and modified by biotic communities (biotic modulation) meet along the way with potentially important implications for downstream community dynamics. In our experiment (b), starting from an initial resource pool (brown circle: standard protist medium, either nondiluted or one‐third diluted), we test the effect of contrasting upstream community structures (descending order from trop: *bacteria alone, monoculture, competition, facilitation,* and *predation*) on bacteria populations in two downstream communities with different trophic structures (one vs. two trophic levels). The two first downstream communities are enlarged to exemplify composition and internal dynamics; analogue settings were present for all downstream systems. B: mixture of three bacteria species (*Serratia fonticola*,* Bacillus subtilis,* and *Brevibacillus brevis*), C_1_: *Colpidium* sp., C_2_: *Paramecium aurelia*, A*: Euglena gracilis*, P: *Daphnia pulicaria,* C_3_: *Tetrahymena pyriformis*

The understanding of directional resource flows is especially relevant for ecosystems or communities in which resource flows are dictated by gravity or dominant wind patterns, such as river ecosystems, mountain slope habitats, or vertically structured plant communities. For example, the “river continuum concept” (Vannote, Minshall, Cummins, Sedell, & Cushing, 1980) suggests that shifts in local community structure along river branches are the sole result of linearly changing physical conditions, and that downstream communities profit from upstream energy processing inefficiencies. A direct but yet unexplored implication of such a directional transfer of energy is that differences in community structure upstream should lead to different downstream dynamics, even in the absence of dispersal of organisms: because biotic interactions modify the way energy is distributed among the different species, the interaction structure of an upstream community should determine the quality and quantity of resources (e.g., dead cells from various species with contrasting stoichiometry and inorganic resources from metabolic waste) flowing through to downstream communities. Therefore, all else being equal, the same amount of resources assimilated by different upstream communities may lead to the production of qualitatively very different subsidies (Gounand, Harvey, Ganesanandamoorthy, & Altermatt, 2017). In a system with reciprocal subsidy exchanges this could alter source–sink dynamics (Gravel, Guichard, et al., 2010) or nutrient colimitation where communities exchange different limiting resources (Marleau, Guichard, & Loreau, 2015). However, in ecosystems with strong directionality, upstream communities are likely to act as mediator of the effects of resource flow on downstream communities (Figure 1a).

As a first demonstration, we here report an experimental test of the effect of upstream community structure on downstream community dynamics in a simplified but highly controlled setting. We addressed potential mechanisms that might control the variable nature of resource‐flux effect such as how varying the upstream community structure will modify upstream resource input into the different downstream communities, and if and how this differently affects their dynamics. Using protist microcosms, we implemented directional flows of nutrients moving from a standard resource pool into upstream communities of contrasting protist interaction structure (“*Monoculture,”* “*Competition,”* “*Predation,”* “*Facilitation,”* “*Bacteria alone,”* see Figure 1b), and then to further downstream communities of either one (bacteria) or two trophic levels (bacteria and a consumer, Figure 1b). We tracked population densities of bacteria and protists in the downstream communities and linked them to the respective upstream community structure.

## 2 METHODS

We studied the effects of directional spatial flows mediated by biotic modulation in sequentially linked communities (called either “upstream” or “downstream,” corresponding to the flow direction, Figure 1). We manipulated the structural composition of the upstream community and monitored subsequent effects on the downstream community in the absence of dispersal of organisms (i.e., only spatial flows of resources).

To test the effect of upstream community structure on downstream community dynamics, we built a factorial protist microcosm experiment composed of 10 types of two‐patch meta‐ecosystems linked by directional spatial flows. Each two‐patch meta‐ecosystem was composed of an upstream community, which was either bacteria alone (a mixture of *Serratia fonticola*,* Bacillus subtilis,* and *Brevibacillus brevis*, referred to as the “*Bacteria alone*” treatment), the same bacteria mixture and the bacterivorous ciliate *Colpidium* sp. (protist “*Monoculture*”), the same bacteria mixture and *Colpidium* sp. with the bacterivorous *Paramecium aurelia* (protist in “*Competition*”), or with the autotroph *Euglena gracilis* (protist “*Facilitation*”, see Figure 1b), or with the generalist predator *Daphnia pulicaria* (protist under “*Predation*”, see Figure 1b). As our focus is on the effect of different upstream community structures on downstream community dynamics, we use only the treatment rather than species names in the text for the sake of clarity and consistency. The choice of each species combination is based on prior knowledge from previous experiments in similar settings (Carrara, Giometto, Seymour, Rinaldo, & Altermatt, 2015; Gounand et al., 2017; Harvey et al., 2016). These five upstream communities were either connected to a downstream community composed of bacteria alone (one‐trophic‐level community) or bacteria with the bacterivorous *Tetrahymena pyriformis* as consumer (two‐trophic‐level community). To test the sensitivity of our results to initial resource concentration and thus the generality of our findings on the effects of upstream community structure on downstream dynamics, we also replicated our experiment with two different initial inflowing resource levels (Figure 1b). To do this, we either did or did not dilute by one‐third of the standard protist medium (Carolina Biological Supply, Burlington, NC, USA, 0.46 g protist pellets 1/L tap water) that was added to the upstream community twice a week (see “Diffusion” section below and Figure 1b). Each of the ten two‐patch meta‐ecosystems was replicated four times for a total of 160 microcosms.

Each microcosm consisted of a 250‐ml Schott bottle that was filled to 100 ml. Microcosms were assembled by first adding 75 ml of pre‐autoclaved and filtered (Whatman filters) standard protist medium, and 5 ml of bacteria inoculum. After 24 hr, to allow time for bacteria growth, we added 20 ml of protist culture with each protist species at carrying capacity (10 ml per species for mixed communities, 20 ml of *Colpidium* sp. for protist *Monoculture* communities, and 20 ml of *Tetrahymena pyriformis* for the two‐trophic‐level downstream communities). Thus, protist communities were added at 20% of their carrying capacity and were allowed to grow 24 hr before the first resource flow event, henceforth referred to as diffusion and described below. In upstream communities with predation, we added five individuals of *Daphnia pulicaria* in each microcosm. For further details on general methods used in our protist microcosm experiments, see Altermatt et al. (2015).

### 2.1 Diffusion

The directional flow of resources from upstream to downstream communities was carried out in three distinct steps to ensure the maintenance of a constant volume in each microcosm. First, 30 ml was removed from each downstream community. Second, 30 ml from each upstream community was sampled and microwaved until boiling to turn all living cells (organisms) into detritus (Harvey et al., 2016). After a 3 hr cooling period at ambient temperature (20°C), the microwaved samples had reached 20°C and were poured into the respective downstream recipient ecosystems. Third, 30 ml of autoclaved standard protist medium (nondiluted or 1/3 diluted according to treatment) was added to each upstream microcosm from the same homogenized medium pool to ensure that effects to downstream communities were not caused by differences in intake resource quality. This manipulation resulted in a directed resource flow from the common resource to the upstream community, and from the upstream community to the downstream community (Figure 1b). Even if all dead bacteria from upstream communities could be not completely lysed by boiling, extensive evidence shows that it does alter cell membrane integrity, potential, and esterase activity significantly enough to leave a clear signature on the flow‐cytometric results (Berney et al., 2008). Because our cell count (gating) was calibrated from previous studies using only living bacteria cells, we are confident that our estimated bacteria density does not include dead bacteria.

Because our main focus was on the mediating effect of upstream community structure on downstream communities via resource flows only, we chose microwaving until boiling as a method to kill living cells, ensuring that no dispersal could occur between our microcosms. While small molecules are likely lysed during boiling, we cannot exclude that other substance than nutrients, potentially acting as kairomones are diffused. Previous work showed that chemical cues from live or dead conspecifics and heterospecifics can be used to inform movement and dispersal decisions (Fronhofer, Klecka, Melián, & Altermatt, 2015; Fronhofer, Kropf, & Altermatt, 2015; Hauzy, Hulot, Gins, & Loreau, 2007) with important consequences for population growth and large‐scale spatial dynamics (Fronhofer, Nitsche, & Altermatt, 2017). However, our main conclusions on the effects of upstream community structure on downstream ecosystem dynamics are consistent with expectations from previous work on nutrient flow effects in similar settings (Harvey et al., 2016). Therefore, we are confident that a majority of the effects we find are due to flows of nutrients. Based on previous work in similar experimental settings, we also know that 30% diffusion represents the best trade‐off to maximize effects of spatial flows while minimizing the mortality effect associated with the procedure (Gounand et al., 2017; Harvey et al., 2016).

### 2.2 Measurements

Measurements were synchronized with diffusion events. The measurements occurred every Monday and Thursday (experimental days 0, 3, 7, 10, and 14, respectively), and diffusion occurred every Tuesday and Friday. At each measurement day, two 0.5 ml aliquots were sampled for each microcosm: one for protist and one for bacteria density analysis. Protist density was measured by using a standardized video recording and analysis procedure (Pennekamp & Schtickzelle, 2013; Pennekamp, Schtickzelle, & Petchey, 2015). In short, a constant volume (17.6 μl) of each 0.5 ml aliquot was measured under a dissecting microscope connected to a camera and a computer for the recording of videos (5 s/video, see Appendix S1 in Online Supporting Information for further details on this method). Then, using the R‐package bemovi (Pennekamp et al., 2015), we used an image processing software (ImageJ, National Institute of Health, USA) to extract the number of moving organisms per video frame along with a suite of different traits for each occurrence (e.g., speed, shape, size) that could then be used to filter out background movement noise (e.g., particles from the medium) and to identify species in a mixture (see Appendix S1). Finally, for bacteria, we measured densities using standard flow cytometry on fresh SYBR green fixated cells using a BD Accuri^™^ C6 cell counter (1/1000 dilution, following protocols in Altermatt et al., 2015).

### 2.3 Statistical analyses

We analyzed effects of directed resource diffusion on downstream population dynamics of bacteria and *Tetrayhmena* separately. To test for the effect of upstream community on downstream community dynamics, we used a three‐way linear mixed effect model (LME) testing the interactive influence of upstream community structure, presence of a second trophic level in downstream community, and continuous time on log‐transformed bacteria density in the downstream communities. In parallel, for *Tetrahymena* (thus only in the two‐trophic‐level downstream communities), we performed a two‐way LME testing for the interactive effects of upstream community structure and continuous time on log‐transformed densities. In both models, to control for temporal pseudoreplication issues, we added replicates and time as nested random factors.

Because we were also interested in linking changes in downstream communities to changes in upstream communities, as a complementary analysis, we also tested differences in bacteria and protist densities among the different upstream community structure treatments. To this end, we used a two‐way LME testing for the interactive effects of upstream community structure and continuous time on log‐transformed densities. We also added replicates and time as nested random factors.

**Figure 2:**
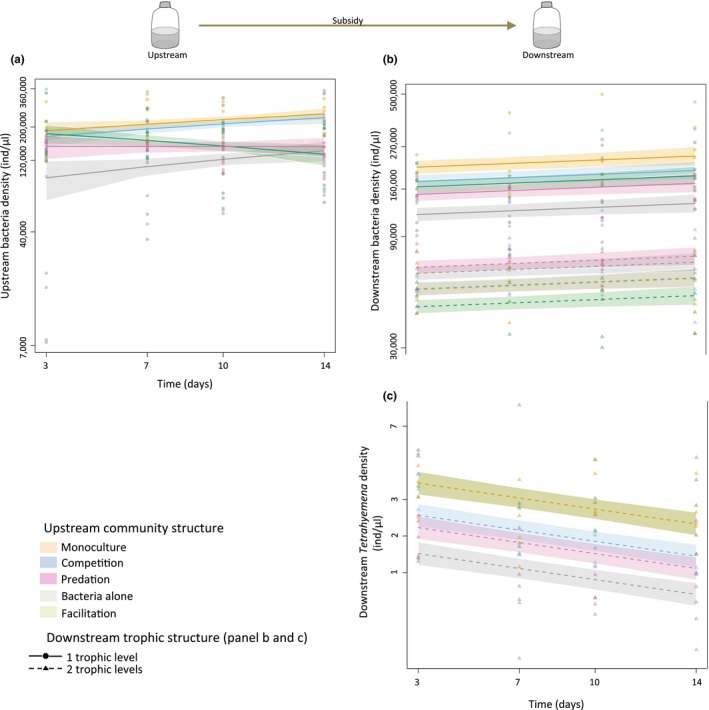
Effect of upstream community structure on upstream bacteria density (panel a), and on downstream bacteria (panel b) and *Tetrahymena* (panel c) densities in the one‐trophic‐level (full lines—*Tetrahymena* absent) and in the two‐trophic‐level (dashed lines—*Tetrahymena* present) communities. Points (*Tetrahymena* absent), and triangles (*Tetrahymena* present) represent raw data. Full and dashed lines represent model predictions with 95% confidence intervals as shadings. Y‐axes on all panels are on log‐scale, but for clarity tick numbers represent raw densities. On panel c, model predictions for *Facilitation* (2.81 ± 1.25 ind/μl) and *Monoculture* (2.76 ± 1.05 ind/μl) are completely overlapped, and on panel b (dashed lines) *Monoculture* (55370 ± 15486 ind/μl) is visible just under *Competition* (55670 ± 17169 ind/μl)

For each LME model, we used an AIC‐based simplification procedure, removing terms sequentially, starting with the highest level of interactions. While we fitted models during model selection using maximum likelihood (“ML”) and the “BFGS” optimization method (Nash, 1990), the final models were refitted by maximizing the restricted log‐likelihood (“REML,” see Pinheiro, Bates, DebRoy, & Sarkar, 2016). We used standardized residuals vs. fitted‐value plots, residual distribution, variance overdispersion, and log‐likelihood information to select the most appropriate transformation for each model. Finally, even if there was not always significant variations over time (i.e., Figure 2b), for the sake of clarity and consistency, we extracted predictions for each LME over time along with 95% confidence intervals, which we report here as our main results (Figure 2). We interpreted treatments with nonoverlapping confidence intervals as significantly different. As complementary information, between treatment differences are reported as mean ± standard deviation in text. The statistical model tables can be readily reproduced by using the provided data and R‐script (https://github.com/harveye/Directional_flow).

All analyses were conducted with R 3.1.2 (R Core Team 2016), using the “bemovi” package (Pennekamp et al., 2015) for video analyses, the “nlme” package for statistical modelling (Pinheiro et al., 2016), and the “car” (Fox & Weisberg, 2011) and “MASS” (Venables & Ripley, 2002) packages to identify proper variable transformations for statistical analyses. All data and the main R‐script to reproduce the results can be downloaded at https://github.com/harveye/Directional_flow.

## 3 RESULTS

**Figure 3:**
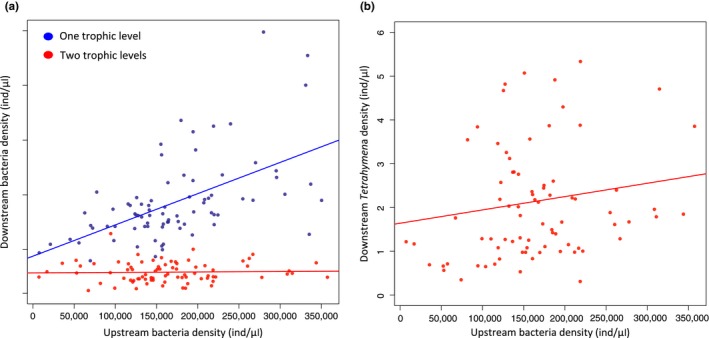
Effect of upstream bacteria density on (a) downstream bacteria density in the absence (blue dots) and in the presence (red dots) of *Tetrahymena* and (b) downstream *Tetrahymena* density. Data points on each panel represent all treatments and experimental days

We examined how downstream communities of varying trophic structure (one or two trophic levels) are influenced by the structure of interactions in upstream communities (see Figure 1b). Changing the dilution factor of the resources flowing through upstream communities led to no qualitative differences in community dynamics; however, in the diluted treatments, population densities of bacteria and *Tetrahymena* were overall two to five times lower, and the range in densities was greatly dampened (for bacteria; between 1400 and 2.4 × 10^5^ ind./μl in the diluted treatment and between 7400 and 5 × 10^5^ ind./μl in the nondiluted treatment, for *Tetrahymena*; between 3 × 10^−3^ and 1.10 ind./μl in the diluted treatment and between 0.3 and 10 ind./μl in the nondiluted treatment, see Figure S1 for paralleled low‐dilution treatment results). Because the results were qualitatively the same, we will further report results only for the nondiluted treatment (but see Figures S1 and S2). As expected, we found that the main driving factor of bacteria populations in the downstream communities was the local presence of a second trophic level (here *Tetrahymena*), which greatly reduced bacteria densities and dampened variation in population densities across treatments regardless of the upstream community structure (CV of bacteria density without *Tetrahymena *= 41.2 and with *Tetrahymena *= 31.2, see Figures 2b and 3a). However, when looking at each downstream community separately, we found significant influences of the upstream communities on bacteria dynamics coupled through resource flows only.

More specifically, in the one‐trophic‐level downstream communities, bacteria density was highest when the upstream community is a *Monoculture* (24 × 10^4^ ± 9.6 × 10^4^ ind./μl) or a *Competition* (20.8 × 10^4^ ± 9.9 × 10^4^ ind./μl, Figure 2b) community, and was lowest when it contained only bacteria (*Bacteria alone*; 13.5 × 10^4^ ± 4.6 × 10^4^ ind./μl, Figure 2b). These patterns matched with bacteria densities in the upstream communities where densities were consistently higher in *Monoculture* (21.6 × 10^4^ ± 6 × 10^4^ ind./μl, Figure 2a) and *Competitive* (20.2 × 10^4^ ± 6.6 × 10^4^ ind./μl, Figure 2a) communities and consistently lower in *Bacteria alone* communities (11.5 × 10^4^ ± 7.7 × 10^4^ ind./μl, Figure 2a). In the upstream *Facilitation* communities, bacteria densities declined over time (Figure 2a) following the expected increase of consumer densities in this treatment (see Figure S2 for *Colpidium* densities in upstream communities). The average bacteria density of 18.1 × 10^4^ ± 3.7 × 10^4^ ind./μl placed this treatment between the highest (*Monoculture* and *Competition*) and the lowest (*Predation* and *Bacteria alone*) treatments, in terms of density, which matched with the pattern observed for bacteria downstream where *Facilitation* also represented the average median density (Figure 2b). In summary, different community structures in upstream habitats supported different levels of bacteria densities, which in turn led to varying levels of detritus transfer (dead bacteria biomass) to the downstream communities, thus influencing their bacteria densities (Figure 2ab).

In the presence of bacterivorous *Tetrahymena* (see Figure 2b), however, bacteria densities did not follow this consistent pattern: highest densities were instead found when the upstream community contained bacteria only (*Bacteria alone;* 6.6 × 10^4^ ± 1.6 × 10^4^ ind./μl, Figure 2b) or a *Predator* (7.1 × 10^4^ ± 1.9 × 10^4^ ind./μl, Figure 2b) and lowest when there was *Facilitation* (4.5 × 10^4^ ± 1.4 × 10^4^ ind./μl, Figure 2b). Bacteria density patterns, in these two‐trophic‐level downstream communities, seem to match local *Tetrahymena* densities with highest bacteria densities found at lowest *Tetrahymena* densities (*Bacteria alone* and *Predation*, Figure 2c) and lowest bacteria densities found at highest *Tetrahymena* densities (*Facilitation*, Figure 2c). Instead of bacteria density (as observed in the one‐trophic‐level treatment, see Figure 3a), it is downstream *Tetrahymena* densities that match with bacteria densities in upstream communities (Figure 3b) with highest densities found for *Monoculture* (*Tetrahymena*: 2.76 ± 1.05 ind./μl, Figure 2c) and lowest densities found for *Bacteria alone* (*Tetrahymena*: 1.25 ± 1.07 ind./μl, Figure 2c). These results suggest varying levels of top‐down pressure from *Tetrahymena* on bacteria in downstream communities as a function of varying upstream community structures.

## 4 DISCUSSION

We experimentally showed that upstream community structure affects downstream community dynamics through resource flows only. Our results demonstrate that different community structures support different bacteria densities in upstream habitats, which in turn lead to varying levels of detritus transfer (dead biomass) to the downstream communities, thus influencing their population densities and trophic interactions in predictable ways (Oksanen, Fretwell, Arruda, & Niemela, 1981). In natural communities with many more species interacting, it is likely that different upstream community structures will also lead to qualitative changes in subsidy depending on the biomass distribution and the respective stoichiometric ratio of each trophic level (Gounand et al., 2017; Marleau et al., 2015; Sitters, Atkinson, Guelzow, Kelly, & Sullivan, 2015). Overall, our work highlights that upstream communities can mediate the effect of resources flow on downstream communities, even in the absence of dispersal.

The presence of a consumer (here *Tetrahymena*) in the downstream communities greatly reduced prey (bacteria) density (see Figure 2). This top‐down pressure varied as a function of upstream community structure: when bacteria density was higher upstream, there were more consumers downstream and less prey, suggesting a spatial cascade through subsidy. Therefore, it seems that the highest trophic level is the most sensitive to changes in resource flow. This finding is consistent with the exploitation ecosystem hypothesis (EEH) proposed by Oksanen et al. (1981), which predicts that increasing nutrient supplies in top‐down‐dominated systems should lead to an increase in consumer (here *Tetrahymena*) but not prey (here bacteria) biomass because of prey regulation by the consumer (see Figure 3). Our finding that bacteria densities are similar under both diluted and nondiluted medium scenarios (Figures 2 vs. S1) because of consumer regulation, but that *Tetrahymena* densities are lower under the diluted scenario because of lower energy supplies are also highly consistent with EEH predictions. Therefore, our result suggests that top‐predators might be key to the response of local communities to spatial variations in subsidy.

In upstream communities, monoculture and competition treatments had the highest levels of bacteria compared to predation and facilitation. Low bacteria density in the predation treatment can be explained by our use of a large generalist predator (*Daphnia pulicaria*) that feeds both on bacteria and protists. In the facilitation treatment, the presence of the autotroph *Euglena gracilis* brings in new resource through photosynthesis that benefits bacteria and likely increases grazing top‐down pressure via a bottom‐up trophic cascade—a pattern that we indeed observed in our results: decreasing bacteria density through time (Figure 2a), paralleled by an increase in the bacterivorous *Colpidium* sp. (Figure S2). These changes to resource quality and quantity may have cascaded to the downstream ecosystems. For instance, we know from previous work with *Daphnia* in similar settings that biomass tends to accumulate at the predator level and thus lead to a decline in detritus quality (increased in recalcitrant chitin content, see Gounand et al., 2017), with negative consequences for connected ecosystems (Gounand et al., 2017). Also, the presence of *Euglena* (facilitation treatment) generates a local enrichment effect, albeit limited by this species slow growth rate (Harvey et al., 2016). Overall, based on our results, downstream community dynamics seem to be mainly driven by variations in upstream bacteria densities (changes to subsidy quantity due to local upstream species dynamics), which likely acted in parallel to changes in resource quality and quantity from other internal dynamics in upstream ecosystems linked to our various community structure treatments (i.e., decreased quality in the *Predation* treatment and increased quantity in the *Facilitation* treatment). Our results thus clarify how upstream communities might affect outflowing subsidy quality/quantity and then cascade spatially to downstream communities. This study also emphasized that measuring detrital content should be a particular concern of future study to further elucidating specific mechanisms.

Interestingly in our upstream ecosystems, we observed that bacteria densities were highest when growing with a consumer (*Colpidium* sp.), and lowest when growing alone. Although this result does not affect our main conclusion that pertains to the matching patterns between upstream and downstream communities as a function of upstream community structure, it is nonetheless a puzzling observation. Although we can only speculate on this, few hypotheses can however explain this counterintuitive result (e.g., selective feeding). Because our bacteria community was composed of three species with wide interspecific size variations, selective feeding by the consumer, releasing one bacteria species from competition, thus leading to higher cell density (but not total biomass), appear to be most likely explanation.

Many natural ecosystems are characterized by directionally biased spatial flows of organisms and resources, such as alpine slopes, seashore habitats, estuaries, vertical structure of tree or plant habitats, and river ecosystems, with the latter likely being the most studied. As opposed to many terrestrial systems, strong directional movements along dendritic‐shaped networks dominate spatial processes in rivers (Altermatt, 2013). These two fundamental attributes of river landscapes (directionality and dendritic‐shaped network) have profound implications for the spatial distribution of diversity and local population dynamics (Carrara, Altermatt, Rodriguez‐Iturbe, & Rinaldo, 2012; Fronhofer & Altermatt, 2017; Kuglerová, Jansson, Sponseller, Laudon, & Malm‐Renöfält, 2014; Seymour, Fronhofer, & Altermatt, 2015; Vitorino Júnior, Fernandes, Agostinho, & Pelicice, 2016). For instance, the river continuum concept (Vannote et al., 1980) suggests that specific communities form in rivers as a function of stream order (i.e., distance to the upstream source). These communities could not be maintained elsewhere because they require the specific physical conditions provided by their location in the river network (Vannote et al., 1980) and recruitment from the directional movement of different upstream organisms from converging paths along the dendritic network (Carrara et al., 2012; Muneepeerakul et al., 2008). Our experiment in a simplified setting successfully disentangled potential mechanisms to explain the upstream–downstream resource coupling. This experimental setting allowed us to single out individual drivers and to address their potential role on ecosystem dynamics. Despite the need for more empirical studies in more complex and natural ecosystems to identify potential contingencies, our experimental results demonstrate that upstream community structure can act as a biotic modulator of resources thus indirectly affecting downstream community dynamics (Figure 1a), with important implications for landscape management and the mitigation of eutrophication issues in downstream habitats.

Our results suggest that upstream species interaction networks might be a key mediator of alterations to downstream habitats in directionally structured ecosystems. For instance, the impact of large nutrient loads from agricultural source upstream on downstream lakes could potentially be mitigated or amplified depending on the interaction structure of upstream communities. In our study, we showed that a specific upstream community structure has the same qualitative effect on downstream dynamics regardless of initial resource concentration (non‐diluted resource; Figure 2 vs. diluted resource; Figure S1). Our experiment thus suggests that biotic interactions *per se* might be a key mediator of spatial changes in community dynamics by indirectly linking communities via directional nutrient flows. This has significant, but yet untested implications for landscape management and the restoration of ecosystem services in ecosystems with directionally biased resource flows.

## CONFLICT OF INTERESTS

None declared.

## AUTHORSHIP

EH, IG, CL, EAF, and FA designed the research; EH and IG conducted the research and processed the data with laboratory support from CL and EAF; EH analyzed the data; all authors participated in results interpretation; EH wrote the first draft of the manuscript; EH and FA edited the first draft; All authors significantly contributed to further manuscript revisions.
