# Supplementary material for: Upstream trophic structure modulates downstream community dynamics via resource subsidies
Source: Ecol Evol. 2017 Jun 15;7(15):5724–31. doi: 10.1002/ece3.3144 (PMC5655794; doi:10.1002/ece3.3144)
Supplement: Supplementary file 1 [file ECE3-7-5724-s001.docx]

Supplementary Material

“Upstream trophic structure modulates downstream community dynamics via resource subsidy.”

by Eric Harvey, Isabelle Gounand, Chelsea Little, Emanuel A. Fronhofer and Florian Altermatt

**Table of contents**

Appendix S1 – protist density counts by video analysis 2

Supplementary figures 4

Figure S1 – Experimental results with diluted resource. 4

Figure S2 – Upstream *Colpidium* density. 5

# Appendix S1 – protist density counts by video analysis

The 5-second videos were recorded with a Canon camera, adjusted on a Nikon microscope with a 20-fold magnification, on 17.6µL-volume samples.

To process the videos, we first detected the moving particles with the functions locate_and_measure_particles(), link_particles() and filter_data() of the R-package bemovi (Pennekamp et al. 2015), coupled to the image analysis free-ware ImageJ (ImageJ, National Institute of Health, USA). The parameters used in the different functions were min_size = 5, max_size = 1000, linkrange = 2, disp = 20, net_filter = 10, duration_filter = 0.1, detect_filter = 0.1, median_step_filter = 3.

The species identification was achieved by comparing the traits extracted from species monocultures to each individual particle detected in videos of species in mixture using the Support Vector Machine algorithm (e1071 R-package, Meyer et al. 2014, function svm()). More specifically:

- to distinguish *Paramecium aurelia* from *Colpidium striatum*, we selected typical individuals in the monoculture with sufficient speed (net_speed > 10 and net_disp > 20) and we eliminated small *Paramecium* (argument: mean_minor > 45) and large *Colpidium* (argument: mean_minor < 35). Then we used the size traits (mean_minor + mean_major) in the model.
- to distinguish *Colpidium striatum* from *Euglena gracilis*, we selected the same *Colpidium* than above in the monoculture and the *Euglena* which were not moving to much, as they typically behave (net_speed < 7). We used all the traits (major_mean, major_sd, minor_mean, minor_sd, gross_speed_mean, gross_speed_sd, net_speed_mean, net_speed_sd, sd_turning_mean) in the model.

To check the probability of species assignation errors of the models, we applied them to the different monocultures. We then used the models on species in mixtures.

We did an additional visual check of the videos to avoid false negative of automated particle detection at low density for *Paramecium* and *Colpidium* (absence / presence records) and we counted the *Euglena* individuals visually on each video because their low speed was leading to systematic underestimations of the density.

# Supplementary figures


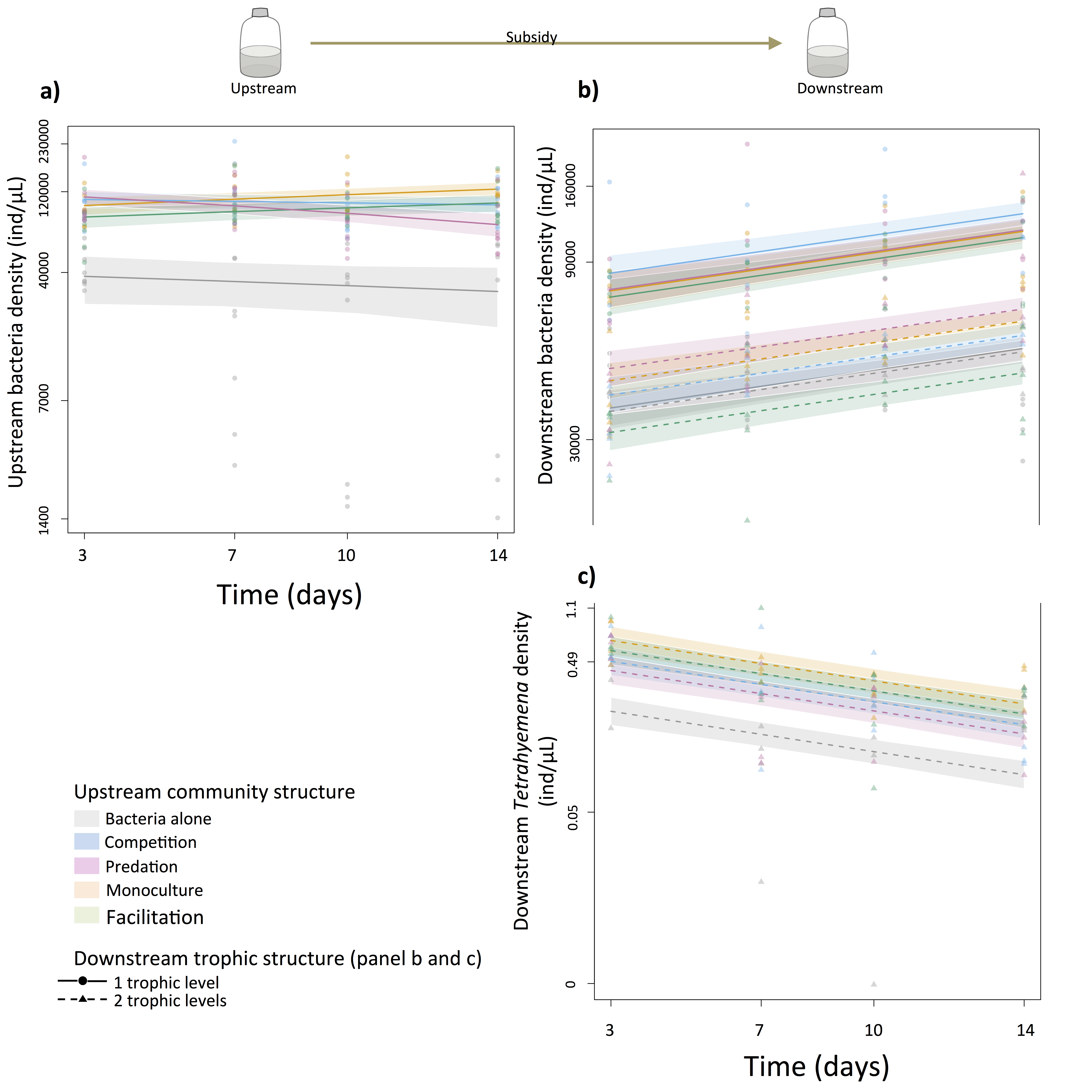


## Figure S1 – Experimental results with diluted resource.

Effect of upstream community structure on upstream bacteria density (panel A), and on downstream bacteria (panel B) and *Tetrahymena* (panel C) densities in the one-trophic-level (full lines – *Tetrahymena* absent) and in the two-trophic-level (dashed lines – *Tetrahymena* present) communities. Points (*Tetrahymena* asbent) and triangles (*Tetrahymena* present) represent raw data. Full lines and dashed lines represent model predictions with 95% confidence intervals as shadings. Y-axes on all panels are on log-scale, but for clarity tick numbers represent raw densities.

## Figure S2 – Upstream *Colpidium* density.

Upstream *Colpidium* density as a function of community structure. Points represent

mean ± se.
